# The Effect of Hepatic Encephalopathy, Hepatic Failure, and Portosystemic Shunt on Brain Volume of Cirrhotic Patients: A Voxel-Based Morphometry Study

**DOI:** 10.1371/journal.pone.0042824

**Published:** 2012-08-13

**Authors:** Long Jiang Zhang, Rongfeng Qi, Jianhui Zhong, Qiang Xu, Gang Zheng, Guang Ming Lu

**Affiliations:** 1 Department of Medical Imaging, Jinling Hospital, Clinical School of Medical College, Nanjing University, Nanjing, China; 2 Department of Imaging Sciences, University of Rochester School of Medicine and Dentistry, Rochester, New York, United States of America; 3 Key Laboratory for NeuroInformation of Ministry of Education, School of Life Science and Technology, University of Electronic Science and Technology of China, Chengdu, People’s Republic of China; Research Inst. of Environmental Med., Nagoya Univ., Japan

## Abstract

**Purpose:**

To evaluate the effect of hepatic encephalopathy (HE), hepatic failure, and portosystemic shunt (PS) on the brain volume alteration in cirrhotic patients with MRI voxel-based morphometry (VBM).

**Methods:**

Sixty cirrhotic patients (overt HE [OHE], n = 11; minimal HE [MHE], n = 19; non HE [nHE], n = 30) including 12 with pre- and post-transjugular intrahepatic portosystemic shunt (TIPS) scanning and 40 healthy controls were recruited. Neuropsychological and laboratory tests were performed in all patients. VBM was analyzed with ANOVA test among 4 groups, and t-tests for patients with different hepatic function, PS scores, and TIPS. Multiple linear regression was performed to investigate the effect of venous blood ammonia levels, Child-Pugh scores, and PS on the brain volumes in all patients.

**Results:**

Cirrhotic patients exhibited decreased volume in many areas of gray matter (GM), increased volume in thalamus, and increased whiter matter (WM) volume, with the extent of affected brain volume greater in HE patients than nHE patients. Hepatic failure also resulted in decreased GM volume. Patients with high PS scores and TIPS displayed decreased GM and increased WM volume in some regions. Post-TIPS patients displayed increased GM volume in the thalamus. Multiple covariate regression results suggested that Child-Pugh score was a major factor to affect GM volume, while PS mainly affected WM volume.

**Conclusion:**

Brain structure abnormalities appeared bilaterally symmetrical in cirrhotic patients, and the impairment was more extensive in HE patients than those without HE. Increased thalamus volume was not associated with HE progression. Hepatic failure and PS altered cirrhotic patients’ brain structure.

## Introduction

Hepatic encephalopathy (HE) is a neuropsychiatric syndrome that develops in patients with severe liver diseases and/or portosystemic shunting (PS) resulted from serious complication of acute and chronic liver failure. HE is characterized by a wide spectrum of clinical manifestations, ranging from alterations of psychometric performance to stupor and coma [1]. Clinically, HE is regarded as a functional disease because it can be reversed after appropriate medical treatments. However, increasing data indicate HE is associated with both functional and structural abnormalities [2], [3].

In last decade, conventional MRI and MR spectroscopy (MRS) have been widely used, which is helpful to uncover the pathophysiological mechanisms of HE [4]. With the use of the blood oxygen level dependent functional MR techniques, brain function impairments and their neural basis, including attention control, brain default mode network, and impaired small-World network efficiency and dynamic functional distribution in the resting state in patients with minimal or overt HE (OHE) have been reported [3], [5]–[8]. Owing to the persisting presence of cirrhosis and portal hypertension, some brain structural abnormalities, such as brain atrophy, can be detected with CT or MRI. Recently, a report with the use of voxel-based morphometry (VBM) measurement of brain tissue volume in patients with or without HE found brain tissue volume loss was common in cirrhosis and progressed during the course of the disease and was greater in patients with history of HE, and persisted after liver transplantation [2]. In addition, symmetric hyperintensity of basal ganglia in T1 weighted MR images is also a common finding in cirrhotic patients, which is regarded as the results of manganese accumulation [4]. Decreased hepatic clearance capacity due to hepatic function failure and the presence of PS in patients with cirrhosis, especially in patients with advanced chronic liver failure, results in manganese accumulation in the basal ganglia. Prior study indicated hyperintensity of globus pallidus on MRI depended on PS [9]. We speculate these comprehensive factors, including HE degree, hepatic failure, and PS, cause the brain volume changes in cirrhotic patients. However, few studies have been reported to systemically evaluate the above-mentioned factors, especially in the PS, on the brain volume changes in cirrhotic patients. The purposes of this study were: 1) to evaluate the brain volume change with HE and hepatic failure progression; 2) to correlate the quantitative PS scores with brain tissue volume measured with VBM in patients with cirrhosis; 3) to investigate the effect of transjugular intrahepatic portosystemic shunt (TIPS) on the brain volume measured with VBM in patients with cirrhosis.

## Materials and Methods

### Participants

This prospective study was approved by our institutional review board and was conducted in compliance with the Health Insurance Portability and Accountability Act. All subjects gave written informed consent before the MRI study. Sixty patients (44 male, 16 female; mean ages 49±10 years) with cirrhosis were recruited for this study. The inclusion criteria for recruitment of the patients were as following: the patients with clinical proven hepatic cirrhosis, who could finish the MR exam without any MRI contraindication, age 18 years or older, and without dental fixtures or other foreign bodies in the head causing significant image artifacts. All the patients had chronic rather than acute liver dysfunction, with complete laboratory tests to evaluate liver function and blood ammonia before MR exam. All patients were right-handed with normal sight. They had no other diseases affecting brain functions, such as drug abuse and trauma. Lactulose was administrated in 11 patients with stage I to II OHE, other medical treatments, such as antibiotics were administrated in almost all of these hospitalized patients with cirrhosis.

Sixteen patients (9 male, 7 female; mean age 51.0±8.8 years) with cirrhosis and portal hypertension scheduled for TIPS in our hospital were included in this study. Covered stent grafts (Fluency stent grafts: 8 mm×6 cm, manufactured by Angiomed GmbHCo. subsidiary of C.R. Bard, Inc. Germany) were used in this study, which were inserted according to standard methods and without complications [10]. Correct stent function was ascertained by the immediate fall in the portosystemic venous pressure gradient and by Doppler ultrasonography.

**Table 1:** Demographics and clinical data of all cirrhotic patients and healthy controls.

Protocols	HC (n = 40)	Patients (n = 60)	*P* value
Gender(M/F)	26/14	44/16	0.37^a^
Age (±SD), y	49.8±11.8	49.7±10.1	0.98^b^
Venous blood ammonia level (umol/L)		58.9±32.3	
Child-Pugh scale (n)			
A		32	
B		23	
C		5	
NCT-A (s)	44.41±9.1	56.74±23.3	0.005^b^
DST (score)	45.00±11.3	33.62±12.6	<0.001^b^
Patients with HE (n)			
OHE		11	
MHE		18	
nHE		31	
Patients with CECT (n)		50	
portosystemic shunt scores ≥4		15	
portosystemic shunt scores <4		35	
Patients with TIPS insertion (n)		12	

Values are expressed as mean ± SD. NCT-A = number connection test type A; DST = digit symbol test; HC  =  healthy control; HE = hepatic encephalopathy; OHE = overt hepatic encephalopathy; MHE = minimal hepatic encephalopathy; nHE = non hepatic encephalopathy; CECT = contrast-enhanced CT; TIPS = transjugular intrahepatic portosystemic shunt.

a The *P* value for gender distribution in the two groups was obtained by Chi-square test.

b The *P* value for age and neuropsychological tests difference between the two groups was obtained by two sample t test.

Forty age- and gender-matched normal subjects (26 male, 14 female; mean ages 49±11 years) without history of psychiatric or neurologic diseases were recruited from the local community. All normal subjects had also no diseases of the liver and other systems. Abdominal ultrasound scans revealed no abnormal findings for all normal subjects.

**Table 2:** Regions showing brain volume differences between OHE patients and healthy controls.

	Brain regions	BA	MNI Coordinates (mm)	Voxel	Peak T value
			(x, y, z)		
GM	ACC	32/24	−2,30,22	96	−3.28
	Right caudate nucleus	25	13,16,7	1027	−5.93
	Left caudate nucleus	25	−7,14,7	856	−6.19
	Right putamen	32/9	9,36,21	459	−4.13
	Left putamen	6	−30,−4,0	567	−5.94
	Left parietal lobule	40/22	−33,−52,55	675	−5.29
	Right middle and inferior frontal cortex	47/10	53,47,3	638	−4.46
	Right superior and middle temporal gyrus	22/21	57,2,−26	2132	−5.80
	Right superior and middle temporal gyrus	22/21	57,2,−26	2132	−5.80
	Right amygdala	36	28,−2,−14	103	−3.97
	Left amygdala	36	−22,−1,−12	71	−3.72
	Cerebellar vermis		−2,−53,−36	285	−3.31
	Right thalamus		15,−26,9	1484	+5.64
	Left thalamus		−9,−20,9	1405	+6.49
WM	Right internal capsule		12,0,−5	579	+6.23
	Left internal capsule		−15,0,−5	821	+6.43
	Right cerebellum crus		21,−50,−40	68	+4.40
	Right external capsule		32,3,−9	319	−3.56
	Left external capsule		−27,0,−2	60	−3.78

Positive sign in the peak T-score represents increase, and negative sign represents decrease in brain volume. All *P*<0.01, FDR corrected.

GM = gray matter; WM = white matter; HE  =  hepatic encephalopathy; BA  =  Brodmann’s area; MNI = Montreal Neurological Institute; ACC  =  anterior cingulate cortex.

### Laboratory Examinations

Blood biochemistry tests, including prothrombin time, protein metabolism tests (including total protein, globulin, albumin, and the ration of albumin and globulin), bilirubin metabolism tests (including total bilirubin, direct bilirubin, and indirect bilirubin), glutamic pyruvic transaminase, and glutamic oxalacetic transaminase, were performed for all patients within one week before MR scanning. All of the above-mentioned tests were used to calculate the Child-Pugh score [11] to assess the severity of liver disease. The score system considered five variables, i.e., ascites, encephalopathy, prothrombin time, and serum levels of bilirubin and albumin, and assigned a score ranging from 1 to 3 to each variable, classifying patients into class A (score 5–6), B (score 7–9) or C (score 10–15). Venous blood ammonia test was obtained in all patients within 24 hours prior to MR scan. No laboratory tests were performed thus unavailable for the 40 normal subjects.

### Neuropsychological Tests

The test battery includes number connecting-A (NCT-A) and digit symbol test (DST), which are recommended by the working party at the 11th World Congresses of Gastroenterology in Vienna in 1998 [11]. The minimal HE (MHE) was diagnosed if a cirrhotic patient showed no clinical overt symptoms of HE but the scores of at least one of the above-mentioned tests were beyond 2 SD (standard deviation) of the mean value for the age-matched controls [12]. According to the West Haven criteria and neuropsychological test results, patients were grouped into OHE, MHE, and non-HE (nHE).

### Portosystemic Shunt Score

All patients underwent dual phase contrast-enhanced CT of the liver at a dual-source CT (SOMATOM Definition, Siemens Medical Solutions, Forchheim, Germany) to evaluate the liver parenchyma and portal vein system prior TIPS, which is a routine procedure in our hospital. Scanning conditions were as follows: slice collimation, 0.6ce4 detectors; slice thickness, 0.75 mm; reconstruction interval, 0.5 mm; helical pitch, 1.4; 120 kVp; 200 mAs. About 70 mL of Omnipaque 300 (Iohexol, Amersham, Shanghai, China) was injected through the right antecubital vein at a flow rate of 3.0–4.0 mL/s. Data acquired at 50 s after injection of the contrast medium were used to reformat portal vein CT images. Reconstructed data were transferred from the scanner to a 3D workstation (Multi Modality Workplace; Siemens Medical Solutions, Erlangen, Germany). The two abdominal radiologists (with 10 years experience in reading abdominal CT images) graded each PS collateral by consensus using a scale system considering total area of shunting collaterals proposed by Yoshikawa et al [13]. Severity was evaluated using a sum of the scores, referred to as shunting collateral score. The cut-off value of 4 was chose according to previous studies [14], [15]; those with a score of 4 or greater were assigned to the group with high-flow PS, and the others to the group with low-flow PS.

### MRI Data Acquisition

MRI data were collected using a 3-Tesla scanner (TIM Trio, Siemens Medical Solutions, Erlangen, Germany). The participants were instructed to keep their heads still during MRI scans. High-resolution T_1_-weighted anatomical images were obtained using a magnetization-prepared rapid gradient-echo sequence (axial, TR/TE = 2300 ms/2.98 ms, flip angle = 9°, 191slices, field of view = 256×256 mm^2^, acquisition matrix = 256×256, slice thickness = 1 mm).

### Data Post-processing

Data analysis was performed using the VBM8 tool-box (http://dbm.neuro.uni-jena.de/vbm) with default parameters running in the SPM8 (statistical parametric mapping, http://www.fil.ion.ucl.ac.uk/spm) software. In detail, structural images were bias-corrected, tissue classified, and transformed into standard Montreal Neurological Institute (MNI) space with a 12-parameter affine-only non-linear transformation, within a unified model. Subsequently, analysis were performed on gray matter (GM) and white matter (WM) segments, which were multiplied by the non-linear components derived from the normalization matrix in order to preserve actual GM and WM values locally (modulated GM and WMvolumes). Finally, the modulated volumes were smoothed with a Gaussian kernel of 8 mm full width at half maximum (FWHM).

### Statistical Analysis

Statistical analysis of structural imaging data was performed using the SPM8 software package. To assess the effect of HE severity on the brain volume in patients, smoothed gray and white matters were analyzed with an ANOVA test to detect regions of brain with volume changes among nHE, MHE, OHE, and control groups. If statistical difference was present, a post hoc *t*-test was performed to detect the inter-group difference of brain regions. The individual’s age and sex were imported as nuisance covariates. Cirrhotic patients were classified into groups with Child-Pugh B+C and with Child-Pugh A since only 5 patients scaled Child-Pugh C. To investigate the effect of hepatic failure and PS on the brain volume, dependent sample *t* tests were used to assess differences in gray or white matter volume between groups with Child-Pugh B+C and Child-Pugh A, and between groups with high-flow and low-flow PS. To evaluate the effect of TIPS insertion on the brain volume, the paired *t* test was used to compare differences in gray matter (GM) or white matter (WM) volume between pre-TIPS and post-TIPS groups.

Considering the inter-effect of PS, liver failure, and venous blood ammonia level on the brain volume in cirrhotic patients, a multiple linear regression analysis was used to investigate the relationship between brain volume in cirrhotic patients and Child-Pugh score, venous blood ammonia level, and PS score in SPM8. A Pearson correlation analysis was performed to study the relationship between brain volume and neuropsychological tests in cirrhotic patients. For the analysis of structural imaging data, statistical threshold was set at *P*<0.01 (after false discovery rate (FDR) correction).

The clinical and biochemical data were analyzed using the software SPSS version 16.0 (SPSS Inc. Chicago, IL, USA) in the study. Dependent sample t test was used to detect the significance for age and neuropsychological tests between cirrhotic patients and controls, while Chi-square test was used to detect gender significance between both groups. *P* values less than 0.05 were regarded as statistically significant.

## Results

### Demographic Data

**Table 1** listed the demographic, clinical and biochemical test data of the cirrhotic patients and healthy controls included in this study. No difference was found for the age and gender between the patients and controls (both *P*>0.05); however, the patients took longer time than controls in performing NCT-A and had lower DST scores than controls (both *P*<0.05). Of 60 patients included, there were 11 patients with OHE, 18 with MHE, and 31 with nHE; 32, 23, and 5 patients were scaled as Child-Pugh A, B, and C, respectively. Ten patients had no abdominal contrast-enhanced CT data to evaluate their PS score; the remaining 50 cirrhotic patients were assessed and subcategorized into high-flow PS in 15 patients and low-flow PS in 35 patients. Of 16 patients with TIPS insertion, 12 patients had available pre- and post-TIPS MR data with a mean 5 months follow-up.

### Brain Volume Change with HE Progression

**Tables 2** **,** **3** **, and** **4** illustrated the brain volume difference between OHE, MHE, nHE patients, and healthy controls. Patients with cirrhosis presented bilaterally symmetrical changes of GM and WM volume. Compared with healthy controls, all cirrhotic patients exhibited decreased gray matter volume in bilateral frontal, parietal and temporal cortex, caudate, amygdale and cerebellar vermis, and increased volume in bilateral thalami. The extent of the affected gray areas was greater in HE patients than nHE patients (**Fig. 1**). WM volume was increased in the internal capsule, cerebellum crus, and decreased in the external capsule. Patients with OHE and MHE demonstrated increased WM volume in the internal capsule than those without HE (**Fig. 2**). From **Table 5** **,** **Figures 1** **and** **2**, in cirrhotic patients, some areas of brain GM and WM volume changes, such as the left inferior parietal lobule, left superior parietal lobule, and left internal capsule aggravate with HE progression from MHE to OHE.

**Table 3:** Regions showing brain volume differences between MHE patients and healthy controls.

	Brain regions	BA	MNI Coordinates (mm)	Voxel	Peak T value
			(x, y, z)		
GM	ACC	32/24	−2,29,25	93	−3.25
	Right caudate nucleus	25	15,16,−2	958	−3.23
	Left caudate nucleus	25	−13,18,−2	875	−3.35
	Right putamen	32/9	9,36,21	439	−3.47
	Left putamen	6	−30,−4,0	524	−4.85
	Right middle and inferior frontal cortex	47/10	38,45,−5	619	−3.53
	Right superior and middle temporal gyrus	22/21	62,−24,−6	871	−4.41
	Left superior and middle temporal gyrus	22/21	−56,−39,1	732	−4.63
	Right amygdala	36	28,−2,−14	115	−4.83
	Left amygdala	36	−22,−1,−12	123	−4.27
	Cerebellar vermis		−2,−64,−29	140	−3.31
	Right thalamus		12,−19,12	1362	+6.41
	Left thalamus		−8,−18,12	1183	+5.29
WM	Right internal capsule		15,3,−2	125	+4.22
	Left internal capsule		−9,0,−2	219	+4.28
	Right cerebellum crus		27,−60,−35	88	+3.74
	Right external capsule		34,4,−2	58	−3.48
	Left external capsule		−30,0,−3	159	−3.27

Positive sign in the peak T-score represents increase, and negative sign represents decrease in brain volume. All *P*<0.01, FDR corrected.

GM = gray matter; WM = white matter; MHE  =  minimal hepatic encephalopathy; MNI = Montreal Neurological Institute; BA  =  Brodmann’s area; ACC  =  anterior cingulate cortex.

**Table 4:** Regions showing brain volume differences between nHE cirrhotic patients and healthy controls.

	Brain regions	BA	MNI Coordinates (mm)	Volume difference (mm^3^)	Peak T value
			(x, y, z)		
GM	Right caudate nucleus	25	15,18,−3	881	−3.19
	Left caudate nucleus	25	−13,15,−2	1016	−3.75
	Right putamen	32/9	12,36,18	775	−3.38
	Left putamen	6	−30,−7,8	585	−4.81
	Right amygdala	36	32,0,−11	64	−3.17
	Left amygdala	36	−21,−2,−12	95	−4.25
	Cerebellar vermis		−2,−64,−29	227	−4.16
	Right thalamus		12,−19,12	1427	+7.83
	Left thalamus		−8,−18,12	1494	+7.58
WM	Right internal capsule		17,2,1	556	+6.69
	Left internal capsule		−12,2,−2	585	+7.20
	Right cerebellum crus		25,−56,−35	108	+4.74
	Right external capsule		34,4,−2	58	−3.48
	Left external capsule		−27,6,−9	160	−3.63

Positive sign in the peak T-score represents increase, and negative sign represents decrease in brain volume. All *P*<0.01, FDR corrected.

GM = gray matter; WM = white matter; OHE  =  overt hepatic encephalopathy; MNI = Montreal Neurological Institute; BA  =  Brodmann’s area; ACC  =  anterior cingulate cortex.

**Figure 1:**
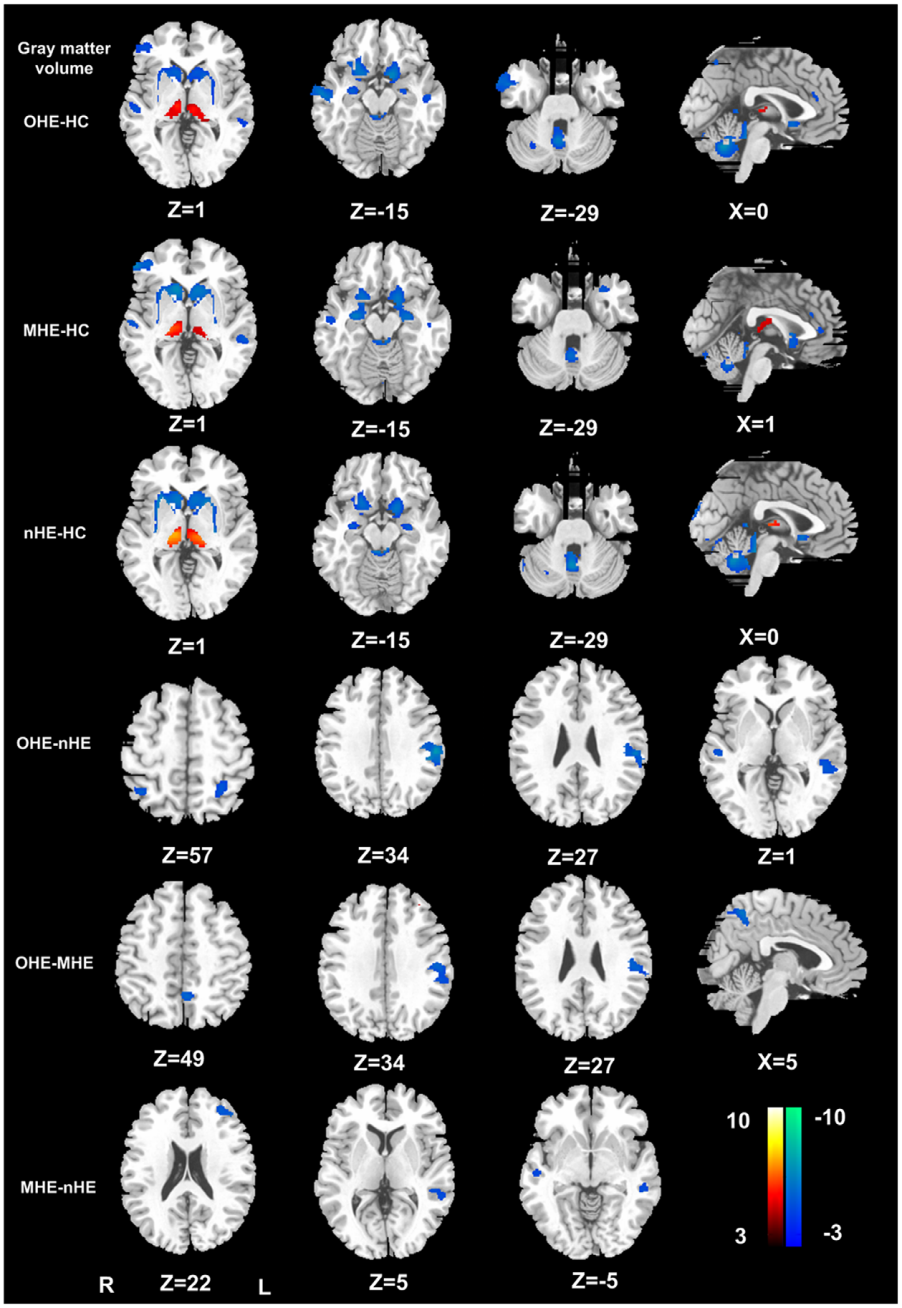
Gray matter changes in healthy controls and cirrhotic patients with different HE stages. Compared with healthy controls, cirrhotic patients exhibited decreased volume in many areas of gray matter, the extension of affected gray areas was greater in HE patients than nHE patients. Increased volume in the thualmus were also found, but was associated with HE progression. (*P*<0.01, FDR corrected). OHE = overt hepatic encephalopathy; MHE = minimal hepatic encephalopathy; HC = healthy controls; nHE = non hepatic encephalopathy; R = right; L = left.

**Figure 2:**
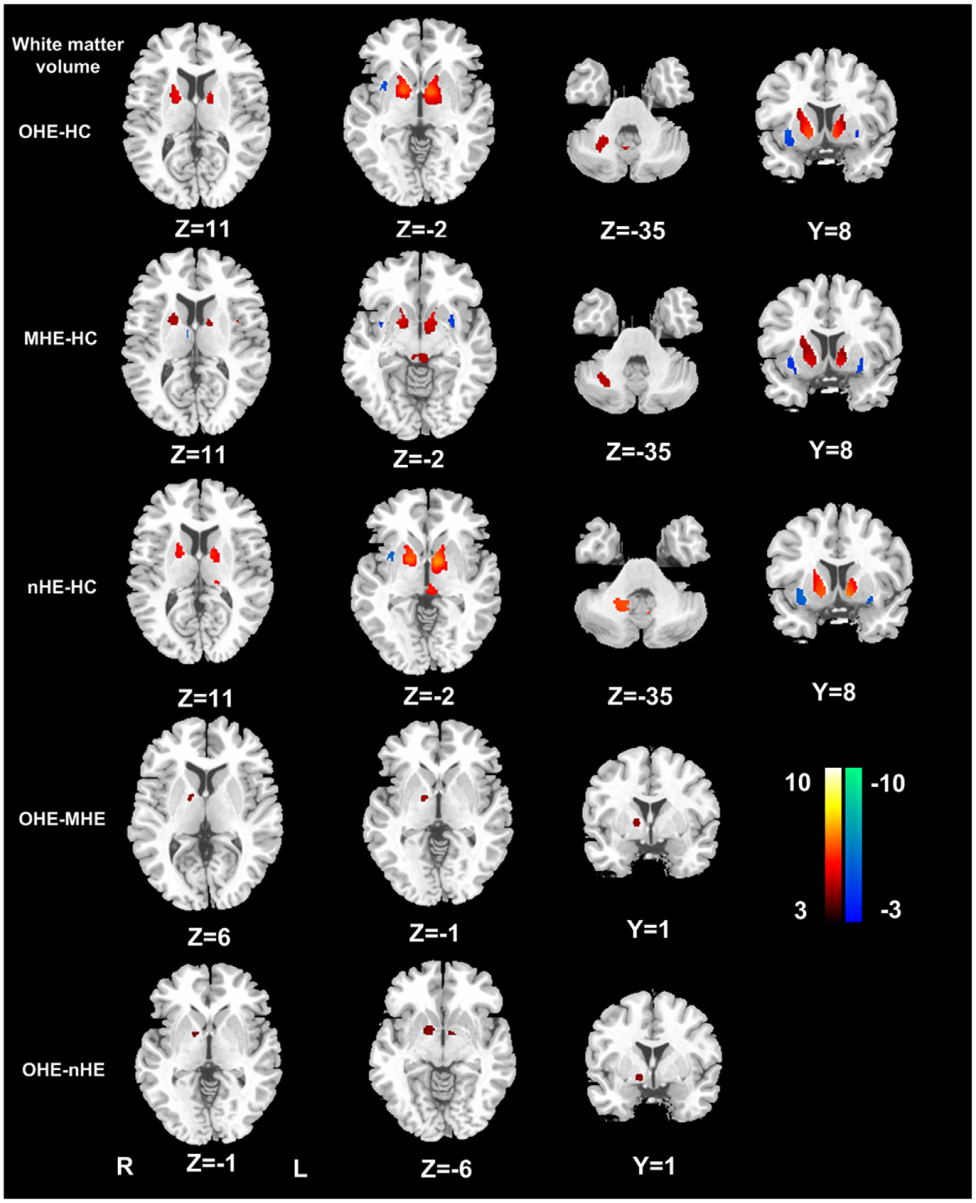
White matter changes in healthy controls and cirrhotic patients with different HE stages. Compared with healthy controls, white matter volume was increased in the internal capsule, cerebellum crus, and decreased in external capsule in cirrhotic patients. Patients with OHE and MHE demonstrated further increased white matter volume in internal capsule than those without HE. (*P*<0.01, FDR corrected). OHE = overt hepatic encephalopathy; MHE = minimal hepatic encephalopathy; HC = healthy controls; nHE = non hepatic encephalopathy; R = right; L = left.

**Table 5:** Regions showing brain volume differences between cirrhotic patients in each stage.

	Brain regions	BA	MNI Coordinates (mm)	Voxel	Peak T value
			(x, y, z)		
OHE-nHE:GM	Right superior and inferior parietal lobule	40/7	42,−49,57	59	−3.12
	Left superior and inferior parietal lobule	40/7	−24,−52,61	644	−3.63
	Right superior and middle temporal gyrus	22/21	59,−16,- 3	1131	−4.31
	Left superior and middle temporal gyrus	22/21	−52,−42,7	1152	−4.68
OHE-nHE:WM	Left internal capsule		−12,2,−2	120	+3.45
OHE-MHE:GM	Left inferior parietal lobule	40/2	−63,−33,33	316	−3.87
	Left superior parietal lobule	7	−27,−52,58	180	−3.40
OHE-MHE:WM	Left internal capsule		−10,2,0	76	+3.25
MHE-nHE:GM	Right superior and middle temporal gyrus	22/21	51,−19,−9	123	−3.74
	Left superior and middle temporal gyrus	22/21	−62,−37,4	392	−4.21

Positive sign in the peak T-score represents increase, and negative sign represents decrease in brain volume. All *P*<0.01, FDR corrected.

GM = gray matter; WM = white matter; MNI =  Montreal Neurological Institute; BA  =  Brodmann’s area; OHE = overt hepatic encephalopathy; MHE = minimal hepatic encephalopathy; nHE = non hepatic encephalopathy; GM = gray matter; WM = white matter.

### The Effect of Child-Pugh Score on Brain Volume

**Figure 3:**
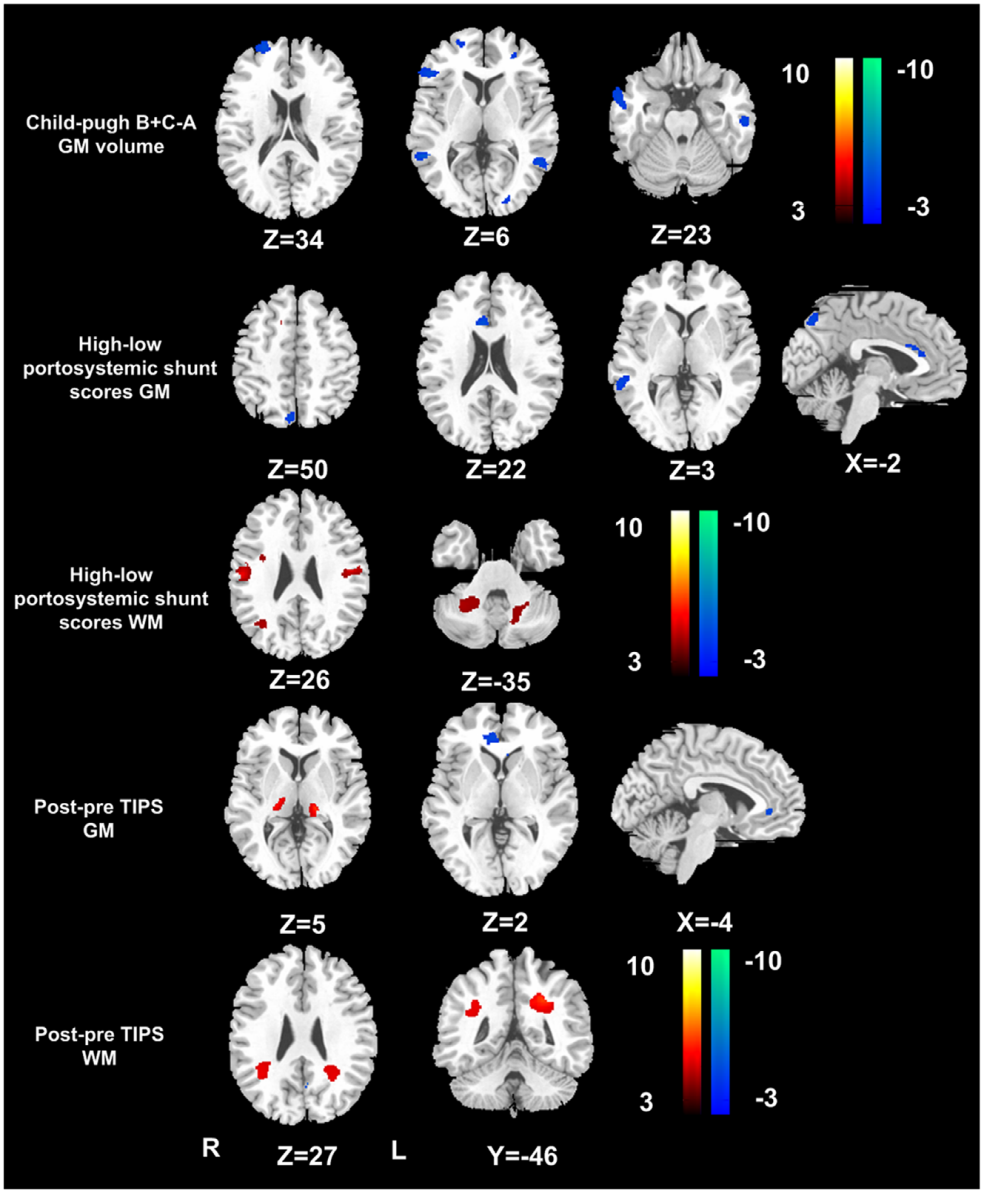
Brain volume changes with Child-Pugh scale and portosystemic shunt. Compared with patients with Child-Pugh A, patients with Child-Pugh B plus C showed decreased gray volume in the frontal, parietal and temporal cortex (Panel at Line 1). Patients with high portosystemic shunt scores (≥4) displayed decreased gray matter volume in the anterior cingulate cortex (ACC) and precuneus, and increased white matter volume in the parietal lobe, internal capsule, and cerebellum crus, when comparing to those with low portosystemic shunt scores (Panels at lines 2 and 3). Compared with pre-TIPS, post-TIPS patients displayed decreased gray matter volume in the ACC, cerebellar vermis, increased gray matter volume in the thalamus, and increased white matter volume in bilateral posterior periventricular white matter (Panels at lines 4 and 5). (*P*<0.01, FDR corrected). GM = gray matter; WM = white matter; TIPS = transjugular intrahepatic systemic shunt; R = right; L = left.

Owing to a limited number of patients with Child-Pugh scale C (n = 5), the patients with Child-Pugh B and C were combined as a group (Child-Pugh B plus C). Compared with patients with Child-Pugh A, patients with Child-Pugh B plus C showed decreased GM volume in the frontal, parietal and temporal cortex (**Table 6** **,** **Fig. 3**), while no WM changes were detected between the two patients groups.

**Table 6:** Regions showing brain volume differences between cirrhotic patients with different clinical markers.

	Brain regions	BA	MNI Coordinates (mm)	Voxel	Peak T value
			(x, y, z)		
Child B+C−A: GM	Right frontal lobe	10/11	19,63,3	961	−3.21
	Right superior temporal gyrus	22	48,−19,−3	92	−3.38
	Left superior and middle temporal gyrus	22/21	−53,−493	317	−3.73
High-low PS scores: GM	ACC	24/33	5,18,27	169	−4.13
	Right precuneus	7	5,−74,49	105	−3.76
	Right middle temporal gyrus	22/21	60,−42,5	196	−4.18
High-low PS scores:WM	Right internal capsule		17,2,1	90	+6.69
	Left parietal regions		−46,−13,27	489	+5.49
	Right parietal regions		46,−12,27	459	+5.21
	Left cerebellum crus		−2,−64,−27	157	+4.87
	Right cerebellum crus		3,−60,−29	162	+3.28
Post-pre TIPS:GM	ACC	24/33	9,39,0	129	−3.85
	Left thalamus		−8,−18,12	336	+5.47
	Right thalamus		12,−19,12	582	+5.04
Post-pre TIPS:WM	Right posterior periventricular white matter		31,−48,30	248	+4.63
	Left posterior periventricular white matter		−25,−44,30	225	+5.97

Positive sign in the peak T-score represents increase, and negative sign represents decrease. All P <0.01, FDR corrected.

MNI =  Montreal Neurological Institute; BA = Brodmann’s area; ACC  =  anterior cingulate cortex; TIPS  =  transjugular intrahepatic portosystemic shunt; PS =  portosystemic shunt; GM = gray matter; WM = white matter.

### The Effect of Portosystemic Shunt on Brain Volume

Patients with high PS scores (≥4) displayed decreased GM volume in the anterior cingulate cortex (ACC) and precuneus, and increased WM volume in the parietal lobe, internal capsule, and cerebellum crus, when comparing to those with low PS scores (<4) (**Table 6** **,** **Fig. 3**).

This study also investigated the effect of TIPS on the brain volume changes in a subgroup of 12 patients undergoing TIPS insertion. Our result showed that compared with pre-TIPS, post-TIPS patients displayed decreased GM volume in the ACC, cerebellar vermis, increased GM volume in bilateral thalami, and increased WM volume in bilateral posterior periventricular white matter (**Table 6** **,** **Fig. 3**).

### Correlation of Brain Volume Changes with Clinical Markers

**Figure 4:**
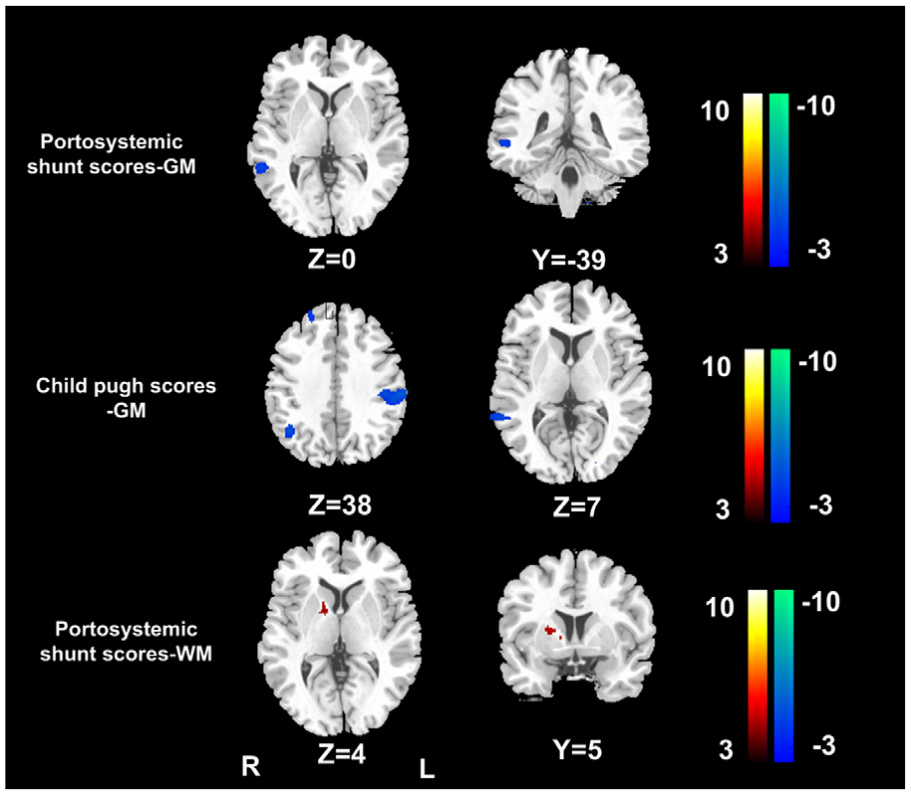
Multiple linear regression analysis between brain volume changes and Child-Pugh scores and portosystemic shunt scores. Multiple linear regression results showed negative correlation between Child-Pugh scores and GM volume of the right superior and middle frontal lobe, right superior and middle temporal gyrus, left inferior parietal gyrus. Negative correlation between PS and GM volume of right middle temporal gyrus and positive correlation between PS scores and WM volume in the right internal capsule were found in cirrhotic patients. (*P*<0.01, FDR corrected). GM = gray matter; WM = white matter; R = right; L = left.

Multiple linear regression results showed negative correlation between Child-Pugh scores and GM volume of the right superior and middle frontal lobe, right superior and middle temporal gyrus, left inferior parietal gyrus (**Fig. 4**). Negative correlation between PS scores and GM volume of the right middle temporal gyrus (**Fig. 4**) and positive correlation between PS scores and WM volume in right internal capsule were found in cirrhotic patients (**Fig. 4**). No correlation was found for the brain volume changes with neuropsychological tests and venous blood ammonia levels in cirrhotic patients (all *P>*0.05).

## Discussion

Our study revealed the following important findings. First, cirrhotic patients had many areas of decreased GM volume and increased WM volume compared with healthy controls which further aggravate with HE progression. Second, hepatic function deterioration was a major factor to affect GM volume in cirrhotic patients. Third, increased bilateral thalami volume occurred in cirrhotic patients, and it was not associated with HE progression but with PS. Forth, PS, either spontaneous or artificial, had an effect on the GM and WM in patients with cirrhosis. To the best of our knowledge, this is the first systemic investigation of various factors on brain volume changes in a relatively large cohort of cirrhotic patients with MRI VBM method.

Our study demonstrated cirrhotic patients had many areas of decreased GM volumes which further deteriorated with HE progression, suggesting selective brain atrophy continuously occurring in cirrhotic patients. The findings have been reported in previous histological, CT and MRI studies [1], [2], [16], [17]. In one recent VBM study [2], Guevara et al. found loss of brain tissue volume progressed during the course of the disease, and it was greater in patients with history of HE. These findings appear to be contradictory to previous MRS studies which found no significant changes of N-acetylaspartate (NAA), a marker of neuron, between cirrhotic patients and healthy controls [18]–[21]. However, many MRS studies placed region of interest in predetermined regions (such as the ACC) [18], [19] or in the WM regions [20], [21], which could miss the change and result in false negative findings. Histopathologically, Alzheimer type II astrocytes in the GM and a patchy but diffuse spongy degeneration of the cortex, characteristic findings in the chronic stage of HE, are responsible for neuronal damage [22]. Being associated with many regions of decreased volume in the frontal, parietal and temporal cortex, caudate and cerebellar vermis, increased WM in many brain areas in cirrhotic patients was also observed, which also progressed during the course of the disease. More recent evidences suggest that there is a synergistic effect between ammonia and various other inflammatory cytokines that result in excess glutamine within astrocytes, leading to osmotic swelling of the astrocytes and the subsequent low grade brain edema as well as other neurocytotoxic effects [23]–[25]. Swelling of the astrocytes secondary to hyperammonia can be a primary cause of increased WM volume in HE. However, decreased external capsule volume was observed but it is difficult to interpret. No correlation was found between venous blood ammonia level and brain volume changes in cirrhotic patients in this study, it is possible that both decreased and increased brain volume in the same patient contributed to this finding. Taken together, it is rather difficult to recognize the brain atrophy because of the coexistence of low-grade brain edema in clinical setting.

One important finding in this study was the observation of increased volume of bilateral thalami in cirrhotic patients compared with healthy controls, even in cirrhotic patients without MHE and OHE. Increased thalamus volume has been reported in patients with history of OHE [3]. However, we found thalamus volume changes occurred not only in the patients with history of OHE but in the patients with MHE and even in the patients without HE. Further subgroup analysis indicated thalamus volume changes only occurred in cirrhotic patients; it is not associated with HE progression. How to interpret this finding is difficult. According to our present knowledge, all information coming from the cortex are passing through the striato-pallidal system indirectly and/or directly back to the thalamus, which might be a filter for sensory inputs [26]. Basal ganglia dysfunction in HE patients, appearing as bilateral hyperintensity in T1 weighted MR images, leads to disinhibition of the thalamus activity, which inputs more inhibitory information back to cortex and results in a series of neurocognitive dysfunctions. Increased volume in the thalamus may indicate neuronal and/or glial hypertrophy or hyperplasia. It is speculated that increased thalamus volume is a compensatory effect for the basal ganglia dysfunction, but increased thalamus volume cannot fully improve brain function. In fact, more other brain areas were involved in the development of HE with disease progression, while the thalamus appears not to be a crucial brain area for HE development.

Hepatic functional deterioration has an effect on the brain volume. Our study found GM volume in regional brain areas further decrease with hepatic failure progression; a multiple covariate regression analysis also showed hepatic failure as a major factor to affect brain volume in cirrhotic patients. However, we did not observe significant WM changes in Child-Pugh B plus C patients compared to patients with Child-Pugh A. This is in contrary to a study by Guevaraet al. [2], where a progression in extent of the affected area was found in WM, and the number of the affected areas also increased with the progression of liver failure. The inconsistent findings between our and the other study may be explained by difference in analysis methods. Our study used a method comparing one subgroup of cirrhotic patients to the other subgroup while Guevara et al.’s study [2] compared the different subgroups of patients with the whole group of healthy subjects. In addition, the difference between the two studies could also come from the small sample size of Child-Pugh C patients included in this study. The current study also showed a negative correlation between Child-Pugh scores and some GM volume in the frontal, occipital, and temporal cortex, which indicted hepatic failure can be an important factor for decreased GM volume in cirrhotic patients. Although a definitive interpretation of these data is impossible, it is perceivable that neurotoxins involved in HE reduce brain volume in areas of GM, which could participate in or predispose to the development of HE.

Portal flow steal is a critical mechanism in the development of HE in cirrhotic patients [1]. The arterial concentration of substances with a high first-pass metabolism, such as ammonia, is highly dependent on portal flow. Based on these facts, we investigated the effect of PS on the brain volume changes in cirrhotic patients. We found patients with higher PS scores had decreased volume in the ACC, right precuneus, and right middle temporal gyrus and increased volume of the right internal capsule, bilateral parietal lobes, and bilateral cerebellum crus than those with lower PS scores. For the patients with TIPS insertion, they had decreased ACC volume and increased bilateral thalami volume and increased volume of bilateral posterior periventricular WM. Importantly, the ACC volume was decreased either in patients with higher PS scores or TIPS insertion. The ACC is regarded as an important core region to attention control, response inhibition, and error detection [27], and it is also a crucial brain region of the default mode network [6], [7], [28]–[31]. In addition, other default mode network areas, such as the right precuneus also was found to have decreased volume in patients with higher PS scores compare to those with lower PS scores. What the default mode network areas represents is not clear completely, but it is known to have a high metabolic activity during rest and is suppressed during cognitively demanding tasks, such as visual and auditory attention, language processing, memory, and motor activities [28]–[31]. Thus, a decreased volume in the above-mentioned brain regions can be involved in the neurocognitive impairment in these patients. In addition to decreased ACC volume, we also found increased thalamus volume in patients with TIPS insertion compared to their pre-TIPS baseline data, which is consistent with the above-mentioned phenomenon observed in cirrhotic patients compared to healthy controls. A compensatory effect of the thalamus in balancing brain function in these patients can be a rational interpretation for this finding [3]. In addition, increased WM volume, although different in brain areas, was consistently found in patients with higher PS scores or with TIPS insertion, which can be attributed to the development of low-grade brain edema [23]–[25]. Taken together, the decreased GM volume and increased WM volume in above-mentioned brain areas can induce chronic HE, while thalamus can be a compensatory role to the basal ganglia dysfunction.

We acknowledge that our study has some limitations. First, the sample size of this study is not large, especially for some subgroups, such as group Child-Pugh C, thus, the statistical power for the subgroup analysis is limited. It is necessary to further collect larger cohort to resolve the issue in future. Second, it is possible that brain volume change is a consequence of liver failure and/or PS with no relationship to HE, while HE is a consequence of brain volume change. This study did not fully uncover the causative correlation of brain volume changes and HE, hepatic failure, and PS. However, we did find above-mentioned factors affect the brain volume in cirrhotic patients. Third, PS scores are evaluated by measuring the diameter of the shunts rather than measuring the blood flow directly, even though the diameter of the shunts reflects blood flow in these shunts to some extent. Fourth, TIPS insertion can have both a short- and long-term effect on brain volume change, but we only observed the brain volume change in a single time point following TIPS. The time-course of brain volume change following TIPS remains unknown. Lastly, lactulose was administrated in 11 OHE patients and other medical treatments in almost all of these hospitalized patients with cirrhosis. These factors can have an effect on brain volume changes in cirrhotic patients; however, it is difficult to exclude these factors because it is necessary to effectively manage OHE patients to deter clinical deterioration.

In conclusion, brain structure abnormalities, i.e., decreased GM volume and increased WM volume, were evident and bilaterally symmetrical in cirrhotic patients, and the impairment was more extensive in patients with HE than those without HE. Hepatic failure and PS further alter cirrhotic patients’ brain structure. Increased thalamus volume was found in patients with cirrhosis and TIPS insertion, which was not associated with HE progression. It is possible that the decreased GM volume and increased WM volume induce chronic HE, while the thalamus plays a compensatory role to the basal ganglia dysfunction.
